# Numerical Simulation and Experimental Investigation of Laser Ablation of Al_2_O_3_ Ceramic Coating

**DOI:** 10.3390/ma13235502

**Published:** 2020-12-02

**Authors:** Shuang Liu, Zongjun Tian, Lida Shen, Mingbo Qiu

**Affiliations:** College of Mechanical and Electrical Engineering, Nanjing University of Aeronautics and Astronautics, No.29, yudao Street, Qinhuai District, Nanjing 210016, China; shuangliu@nuaa.edu.cn (S.L.); ldshen@nuaa.edu.cn (L.S.); qiumingbo@nuaa.edu.cn (M.Q.)

**Keywords:** laser ablation, heat transfer, laminar flow, tensile stress, level set method, Al_2_O_3_ ceramic coating

## Abstract

This paper presents an evaluation of the molten pool laser damage done to an Al_2_O_3_ ceramic coating. Mechanism analysis of the laser damage allowed for a 2D finite element model of laser ablation of the Al_2_O_3_ ceramic coating to be built. It consisted of heat transfer, laminar flow, and a solid mechanics module with the level set method. Results showed that the laser damage mechanisms through laser ablation were melting, gasification, spattering, and micro-cracking. The ablation depth and diameter increased with the increasing laser ablation time under continuous irradiation. The simulation profile was consistent with the experimental one. Additionally, the stress produced by the laser ablation was 3500–9000 MPa, which exceeded the tensile stress (350–500 MPa), and fracturing and micro-cracks occurred. Laser damage analysis was performed via COMSOL Multiphysics to predict laser damage morphology, and validate the 3D surface profiler and scanning electron microscope results.

## 1. Introduction

In recent years, the rapid development of high-energy laser weapons has meant that aircraft and space vehicles are threatened by laser weapons [1]. When a laser irradiates the coating surface of the spacecraft, part of the laser energy is reflected, and the rest of the energy is absorbed by the coating and converted into heat energy, making the internal energy of the spacecraft shell increase, and the temperature rises rapidly until ablation damage occurs [2]. Therefore, the study of laser irradiation characteristics of spacecraft coatings needs to be a focus of research. 

When the laser beam irradiates the target continuously, the thermal accumulation effect will cause ablation damage to the substrate materials. Moreover, a solid-state laser device has a good beam quality, making the beam energy relatively concentrated in the transmission process [3,4,5]. The irradiation area power density is about as high as 103–105 W/cm^2^. Intense ablation can easily penetrate an aircraft shell. Therefore, to prevent laser damage, application of protective material is essential. Currently, the main protection method is preparing various functional coatings to apply on the shell surface. 

When the laser intensity is high enough, the irradiated material surface will undergo complex physical processes, such as melting, vaporization, and even ionization that forms a plasma. Observing and measuring these processes in an experiment is difficult, and the cost is expensive, especially for the high-power laser and materials. Therefore, studying the simulation of laser interaction with matter is necessary. In 1987, Chan and Mazumder [6] proposed a one-dimensional steady-state model considering the phase transformation mechanism, which can describe the one-dimensional temperature field caused by laser thermal damage to materials. In 1990, Kar and Mazumder [7] developed a two-dimensional model to study the melting and vaporization breaking effect on materials during laser irradiation. In 2005, based on the one-dimensional temperature model, Zhang et al. [8] studied the ablation characteristics in detail, considered the dynamic boundary and dynamic absorption rate changes with time under the laser ablation material, and described the physical mechanism of the laser ablation process. In 2011, Li and Lao et al. [9] established a 3D temperature model to investigate femtosecond ablation on aluminum film. It presented the energy conversion process during femtosecond ablation. Using the model, 3D ablation pits were obtained under different energy single-pulse irradiation bursts, and the pit depth and radii before ablation could be predicted conveniently and accurately. In 2017, Wang and Shen et al. [10] established a finite element model of the pulsed laser ablation of aluminum, accounting for the instantaneous removal of materials during the laser ablation process. The numerical simulation model not only predicted the surface degradation caused by material evaporation in the low-laser flux area but also captured the ablation depth caused by the phase explosion in the high-laser flux area. In 2019, Yan and Mei, et al. [11] studied the nanosecond laser ablation process of titanium at 1064 nm and proposed a multi-physical axisymmetric two-dimensional model. The surface morphology was cratered after ablation, and increasing laser flux led to the slow nonlinear growth of ablation depth and melting zone diameter. 

The Al_2_O_3_ ceramic material has excellent high-temperature resistance [12]. It can effectively prevent high-temperature substrate damage by dissipating the laser and blocking heat [13,14,15]. The laser ablation effect is important in researching and developing protective coatings. Through laser irradiation experimentation, the protective effect of the prepared material samples is verified. 

In this paper, according to the current research needs regarding the aviation laser protective material Al_2_O_3_, an ablation experiment platform was built based on a high-power continuous solid-state laser. Moreover, comprehensive research work, including a theoretical simulation and experiment, was carried out. In this paper, a two-dimensional model was established by considering the mechanisms of energy absorption and heat conduction, latent heat of phase change, thermal stress, and thermal physical parameters of materials. The dynamic distribution of the morphology of laser ablation was simulated. The simulated and experimental results of the physical processes of laser ablation are reported. 

## 2. Model Description

### 2.1. Physical Model

**Figure 1:**
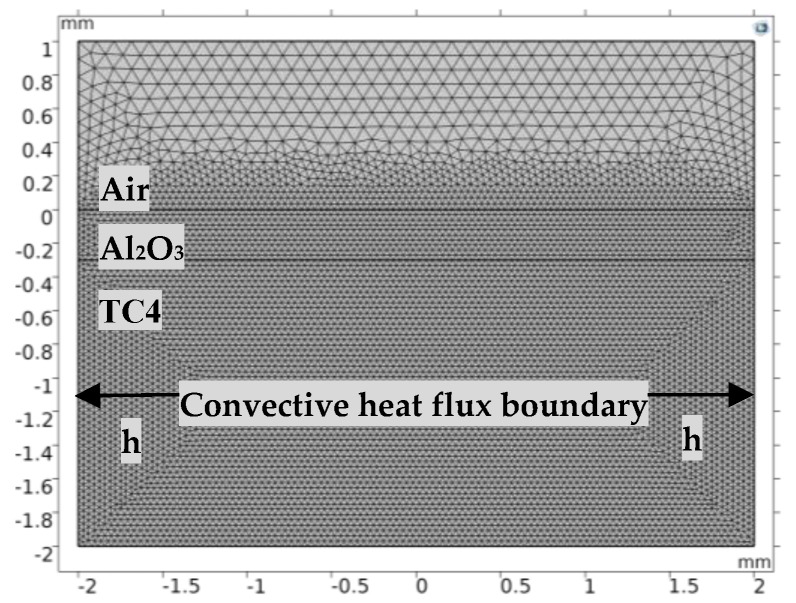
2D model and meshing of sample.

According to laser irradiation characteristics, utilizing COMSOL Multiphysics 5.5, a two-dimensional finite element model was established. The COMSOL Inc. was founded in Stockholm, Sweden. The calculation area was 4 mm in the x-direction and 3 mm in the y-direction, as shown in Figure 1. The occupied air space was 1 mm on the top, that of the Al_2_O_3_ ceramic material was 300 μm, and that of the TC4 material ((Ti-6Al-4V)) was 1.7 mm. A Gaussian laser source was applied to the top surface to simulate its propagation in the y-direction. Zero flux boundary conditions were applied at the bottom. The convective heat flux boundary conditions were applied on the left side and right side. The initial condition was 293.29 K ambient temperature and 1 bar ambient pressure. To simplify the calculation, the following assumptions were made:

- The liquid ceramics flow in the molten pool was considered as an incompressible Newtonian laminar flow.
- The surrounding air was regarded as incompressible airflow.
- Ceramic vapor was considered an ideal gas and transparent to the incident laser beam.
- The formation of plasma was not considered.
- Multiple reflections were not considered.
- The boiling point of ceramics was independent of pressure.
- There is no melting of the substrate during laser ablation, heat transfer is considered only.

**Figure 2:**
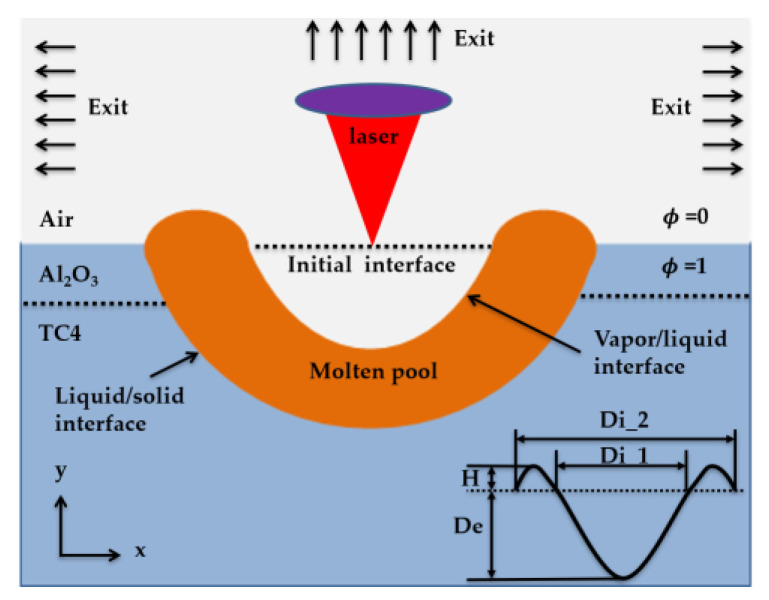
The schematic of simulation model for laser ablation.

The laser ablation simulation model schematic can be seen in Figure 2. Di_1 and Di_2 were the inner diameter and outer diameter of the laser damage morphology, respectively; H and De were the laser damage morphology height and depth, separately. The detailed content is shown in Section 4.1.1 and Section 4.1.2.

### 2.2. Heat and Fluid Flow Model

When the laser beam irradiated the Al_2_O_3_ ceramic coating surface, the target and surrounding environment were heated via heat conduction, convection, and radiation. With the temperature increasing, under the action of material removal and recoil pressure in the laser energy absorption process, the melt vaporized, spouted, boiled, and locked. To explain the interaction mechanism between the laser and the Al_2_O_3_ ceramic coating, the general form of conservation equations of mass (continuity) (1), momentum (Navier-Stokes) (2), and energy (heat) (3) could be used in the whole calculation field.

(1) $$\nabla \vec{u}=0$$

(2) $$\begin{array}{l} \rho \left(\frac{\partial \vec{u}}{\partial t}+\vec{u}\cdot (\nabla \cdot \vec{u})\right)=\nabla \cdot [-pI+\mu (\nabla u+(\nabla \vec{u}))]\\ +\rho \vec{g}+F+F_{\delta } \end{array}$$

(3) $$\rho C_{p}(T)\left[\frac{\partial T}{\partial t}+\nabla \cdot (\vec{u}T)\right]=\nabla \cdot (k(T)\nabla T)+Q_{L}$$

$C_{p}$ was the specific heat capacity of materials ((Equation (16) and Table 1) and $\vec{u}$ was the molten velocity, p was the pressure, ρ was the density of the material (Table 1 and Table 2), I was the identity matrix, $\vec{g}$ was the gravity acceleration, and *T* was the absolute temperature, F was the body force. $F_{\delta }$ was the surface tension force. k was the thermal conductivity of materials ((Equation (18) and Table 1)). $Q_{L}$ was the laser source term. 

### 2.3. Modified Level Set Method

In the above expressions (1 to 3), the laser parameters and the thermophysical properties of the Al_2_O_3_ ceramic coating irradiated by the laser included solid, liquid, or gas (Table 2). Solids and liquids were controlled via temperature, and liquids and gases were controlled via level sets. The buoyancy effect was ignored only in the vaporization due to the high steam velocity, and the Marangoni effect was considered. 

To track dynamically the interface between liquid and gas, the level set method [16] was utilized. 

For the Al_2_O_3_ ceramic: 

ϕ = 1;

For the air: 

ϕ = 0;

To simulate the vaporization and back stamping mechanism generated in the laser irradiation process, the technology in [17] was used in this paper. This technology corrected the mass conservation equation. In Equation (4), the term on the right side of the equal sign is only non-zero on the interface, and this is only the case when the temperature is higher than the vaporization temperature. This item was based on the mass-loss rate [18] generated by the Knight formula-generated transformation evaporation. When both conditions were not satisfied, this equation was equivalent to the classical conservation equation of mass that was seen earlier. According to the level set method transfer equation (Equation (5) [16]) of the above formula, corresponding modifications were made to represent correctly the boundary mass transfer by adding a term that was dependent on the mass flow [18]. 

At the interface, the energy balance was modified when vaporization occurred. Considering the latent evaporation heat, Equation (6) was adopted [19] and the condensation phenomena were neglected.

(4) $$\nabla \vec{u}=\dot{m}\delta (\phi )\left(\frac{\rho_{l}-\rho }{\rho^{2}}\right)$$

(5) $$\frac{\partial \phi }{\partial t}+\vec{u}\cdot \nabla \phi -\dot{m}\delta (\phi )\left(\frac{1}{\rho }\right)=\gamma_{ls}\nabla \cdot \left(\varepsilon_{ls}\nabla \phi -\phi (1-\phi )\frac{\nabla \phi }{|\nabla \phi }\right)$$

(6) $$p_{sat}(T)=p_{a}\exp\left[\frac{\Delta H_{v}}{k_{b}T_{v}}\left(1-\frac{T_{v}}{T}\right)\right]$$

(7) $$\dot{m}=\sqrt{\frac{m}{2\pi k_{b}}}\frac{p_{sat}(T)}{\sqrt{T}}\left(1-\beta_{r}\right)$$

(8) $$Q_{v}=-L_{v}\dot{m}\delta (\phi )$$

where $\gamma_{ls}$ was the level set interface re-initialization velocity value, $\Delta H_{v}$ was the phase change enthalpy, $\varepsilon_{ls}$ was the transition thickness, $k_{b}$ was the Boltzmann constant (Table 2), $p_{sat}(T)$ was the saturated vapor pressure, $\beta_{r}$ was the retro-diffusion coefficient, $\dot{m}$ was the atomic weight of the material, and $L_{v}$ was the latent heat of vaporization. $Q_{v}$ was the evaporation capacity. δ(ϕ) was the delta function of the level set value. 

### 2.4. Phase Transition

During laser processing, the irradiated zone experienced phase changes of melting and vaporization. To deal with the latent heat from the phase transitions, an equivalent specific heat capacity method was applied to the model, which can be given as [20,21,22]:

(9) $$C_{p}^{0}=C_{p}+L_{m}D_{m}+L_{v}D_{v}$$

(10) $$D_{m}=\frac{\exp\left[-{\left(T-T_{m}\right)}^{2}/\Delta T_{m}^{2}\right]}{\Delta T_{m}\sqrt{\pi }}$$

(11) $$D_{v}=\frac{\exp\left[-{\left(T-T_{v}\right)}^{2}/\Delta T_{v}^{2}\right]}{\Delta T_{v}\sqrt{\pi }}$$

With the laser power density increased, the irradiated zone temperature exceeded melting point Tm and boiling point $T_{v}$ successively. When $T<T_{m}$, the Al_2_O_3_ ceramic coating absorbed the laser energy. When $T_{m}<T<T_{v}$, mass flow entered the liquid ceramic at a different speed, and evaporation led to mass removal when $T>T_{v}$.

### 2.5. Laser Heat Source

In the laser irradiation process, the phenomena of evaporation, convection, and surface radiation to the environment, which caused part of the heat to be lost, would occur. Therefore, the following formula was obtained [23]:

(12) $$k\nabla T=AQ_{L}-\left[h\left(T-T_{0}\right)+\varepsilon k_{b}\left(T^{4}-T_{0}^{4}\right)\right]$$

where A was the absorptivity of the Al_2_O_3_ ceramic on the 1064 nm laser, h was the heat transfer coefficient, $k_{b}$ was the Boltzmann constant (Table 2), ε was the surface emissivity (Table 2), and the thermal insulation condition, k∇T = 0, was applied on other boundaries. The initial temperature was $T_{0}$ = 293.29 K.

$Q_{L}$ was the laser heat flux with the Gaussian distributed laser energy acting on the interface between the air and the Al_2_O_3_ ceramic, which could be expressed as [24]:

(13) $$Q_{L}=\frac{2Q}{\pi r_{—}spot^{2}}\exp\left(\frac{-2x^{2}}{r_{—}spot^{2}}\right)$$

Q was the initial laser power, $r_{—}spot$ was the laser spot radius.

The laser on and off control equations were expressed by a step function. If the laser was on, the *L*(t) = 1, otherwise the *L*(t) = 0. The equation is as shown:

(14) $$L(t)=0,\;t\geq t_{\mathrm{on}}$$

(15) $$L(t)=1,\;t\leq t_{\mathrm{on}}$$

where $t_{\mathrm{on}}$ was the laser emission time. 

Material properties are assumed temperature dependent as given by Equations (16) [25], (17) [25], (18) [25], (19) [26], (20) [26].

(16) $$\begin{array}{l} C_{p}(\mathrm{kJ}/\mathrm{kg}*K)=-40.92+(4.022\times T)-\left(5.0048\times 10^{-3}\times T^{2}\right)\\ +(5.681\times 10^{-6}\times T^{3})-\left(6.2488\times 10^{-10}\times T^{4}\right) \end{array}$$

(17) $$\alpha \left(K^{-1}\right)=[-0.23036+\left(7.0045\times 10^{-4}\right)\times T+\left(5.681\times 10^{-8}\right)\times T^{2}]\times 10^{6}$$

(18) $$\begin{array}{l} k\left(W/(m*K)\right)=85.868-0.22972T+2.607\times 10^{-4}T^{2}-1.3607\times 10^{-7}T^{3}\\ +2.7092\times 10^{-11}T^{4} \end{array}$$

(19) $$E(\mathrm{GPa})=407.10-7.3407\times 10^{2}T$$

(20) $$\gamma (N/m)=0.64-8.2\times 10^{-5}\left(T-T_{m}\right)$$

## 3. Experimental Details

In this preliminary study, the raw material used in the experiment was Al_2_O_3_ ceramic powder (purity 99.99%, particle size 15–50 μm). It was provided by Hangzhou Wanjingxin Material Co. Ltd. The Al_2_O_3_ ceramic coating with a thickness of 300 μm on the TC4 alloy surface was prepared via thermal spraying [31,32], and a sample with a size of 4 × 4 × 1.7 mm^3^ was obtained. The experiments were conducted on laser equipment, which included a 4 kW FEIBO fiber laser, and a KUKA robot working table. The laser ablation process was conducted on the square samples with a power of 500 W and an irradiation time of 25 ms. After the laser perforation experiment was conducted, the micromorphology and microstructural characteristics were observed by a scanning electron microscope (SEM). The equipment model was the ZEISS Sigma 500 (CARIZEISS, Oberkochen Germany). COMSOL 5.5 software was used to simulate the laser irradiation topography evolution. The laser damage profile was characterized by a 3D surface profiler (model: NanoMap-1000WLI). The laser experiment parameters are given in Table 3. 

## 4. Results and Discussion

The laser ablation process was simulated by comparing the experimental results with the simulation results, analyzing the evolution and formation mechanism of laser damage morphology, and verifying the model validity. Moreover, the surface tension effect on the molten morphology and the impact of the temperature field, flow field, and stress field on melting and vaporization were discussed. 

### 4.1. Experimental and Numerical Results Comparison

#### 4.1.1. Temperature Field, Fluid Flow and Laser Damage Morphology

**Figure 3:**
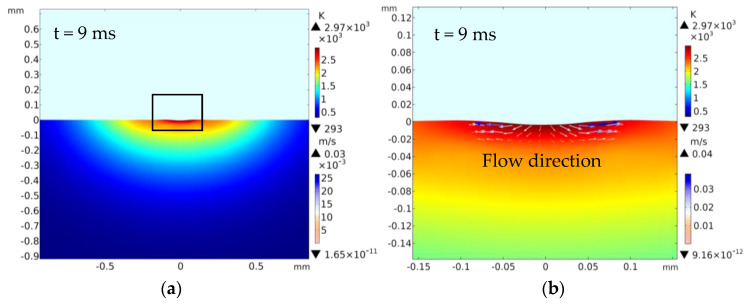

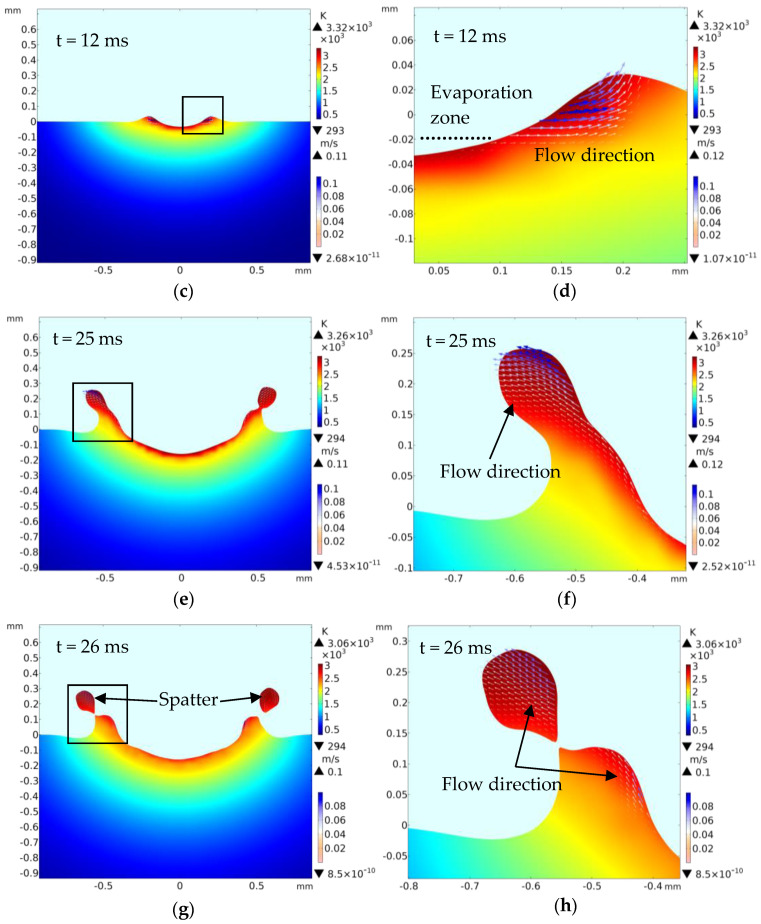
Simulation of temperature distribution, flow distribution, and morphology evolution: (**a**) t = 9 ms, (**c**) t = 12 ms, (**e**) t = 25 ms, (**g**) t = 26 ms; (**b**,**d**,**f**,**h**) are the amplification micrograph at the black frame of (**a**,**c**,**e**,**g**), separately.

**Figure 4:**
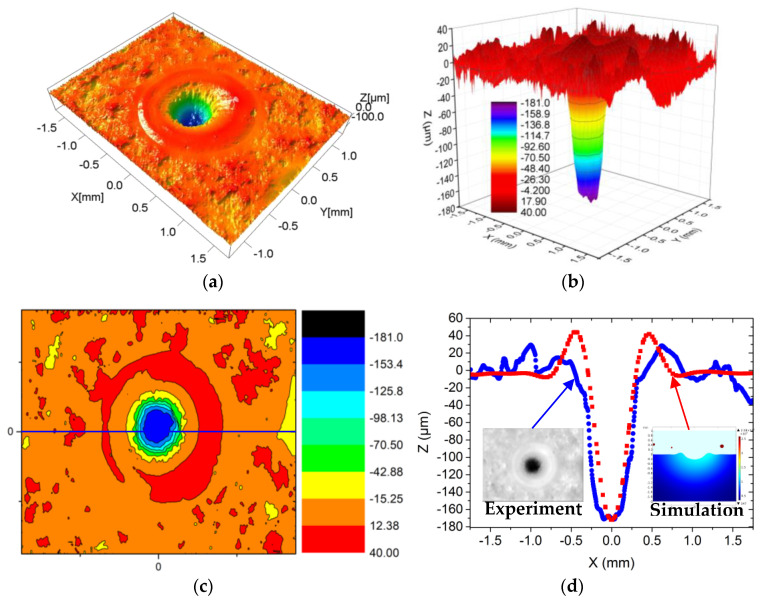
Comparison of laser damage profile between the experimental and simulation-based results: (**a**) three-dimensional scanning inspection map; (**b**) the color map surface drawn by Origin; (**c**) the contour profile drawn by Origin; (**d**) the comparison between the experimental 2D cross-sectional profiles and the numerical 2D cross-sectional profiles at 56.9 ms.

A laser power of 500 W for the laser ablation processing is shown in Figure 3a,c,e,g, which presents the melting and vaporization evolution process. Figure 3b,d,f,h shows the amplification micrograph at the black frame of Figure 3a,c,e,g separately, which demonstrates the fluid flow speed with different laser ablation time. A comparison of the laser damage profile between the experimental and simulation-based results is shown in Figure 4a–d, and the corresponding laser ablation times are each 9, 12, 25, and 26 ms (t > t_on_). The four experiments are detailed as follows. 

**Figure 5:**
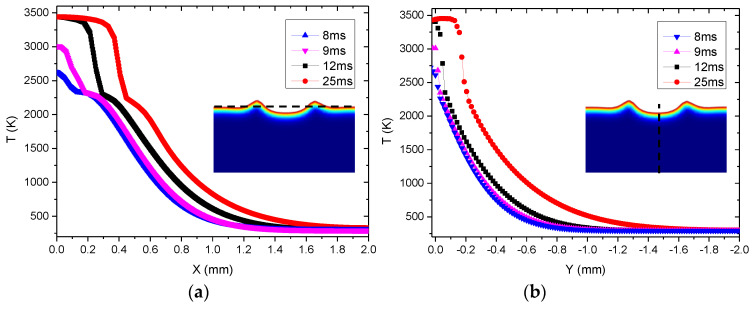
Temperature distribution curves with laser ablation time: (**a**) horizontal (black dotted line position); (**b**) longitudinal (black dotted line position).

When the laser ablation time was 9 ms, Figure 3a,b shows that the center position (Di_1: −0.05~0.05 mm) appears to be a little sunken. The reason was that the temperature exceeded the melting point (T_m_) of the material, which was in line with the temperature field distribution in Figure 5. The material had melted. At this time, the laser damage began to occur. Figure 3b shows that bulges like hills around the ablation pit fringe appeared, with heights of 1 um and 1.5 um. Due to the Marangoni effect driven by a surface tension gradient, the solution flowed from the center up the sides. The flow speed was approximately 0.01~0.04 m/s (Figure 3b). 

When the laser ablation time was 12 ms, a part of the material in the center had evaporated and been consumed simultaneously. Compared with the above content (t = 9 ms), the diameter and depth of the laser damage increased rapidly (Di_1: −0.15~0.15 mm), and the laser damage morphology became clearer with the time increasing (Figure 3c). In addition, Figure 3d shows that bulges like hills around the ablation pit fringe increased, with heights of 30.3 μm. With time increasing, the melting region increased and evaporation zone was about −0.08~0.08 mm. Due to the Marangoni effect driven by a surface tension gradient and reverse stamping effect driven by gasification, the liquid flowed upward (Figure 3d); the maximum flow speed was approximately 0.12 m/s (Figure 3d). This produced laser damage with a “sine curve” appearance. 

When the laser ablation time was 25 ms, the high temperature above the vaporization temperature caused the Al_2_O_3_ ceramic material to be removed via vaporization, resulting in a concave shape. With the irradiation time increasing, and much more material being removed, the depth and diameter increased as a result (as shown in Figure 3e). When the time was 26 ms (the laser on time 25 ms, the laser off time 1 ms), the spatters occurred as shown in Figure 3g,h. Under the combined action of recoil pressure and surface tension, the molten solution flowed upward. When the vertical momentum was greater than the surface tension, the molten ceramic at the edge separated from the molten pool to form spatter [22]. The spatters can be observed in Figure 9. 

**Figure 6:**
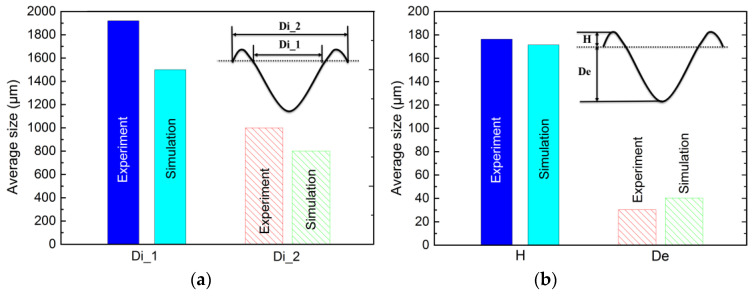
Comparison of the data between experimental results and simulation results of the laser damage profile: (**a**) numerical value of inner (Di_1) and outer (Di_2) diameter of damaged hole; (**b**) numerical value of height (H) and depth (De) of damaged hole.

Figure 4a–d shows the laser damage profile of the experiment and the simulation (t = 59.6 ms, the laser ablation time 25 ms, the solidification time 34.6 ms). Figure 4a shows the experimental three-dimensional image. Figure 4b,c shows the color map surface and the contour profile drawn using the Origin software, respectively. Figure 4d shows that the comparison between the experimental 2D cross-sectional profiles and the numerical 2D cross-sectional profiles at 56.9 ms. As shown in Figure 4d, the laser damage profile is consistent with the simulation result. For a better comparison, Figure 6 shows the experimental and simulation data of the laser damage profile. The damage hole diameters in the experimental and the simulation results were each approximately 1910.54 μm (Di_1), 1580.23 μm (Di_1), and 1002.25 μm (Di_2), 803.63 μm (Di_2). The hole depth (De) in both was 175.21 μm and 171.71 μm (Figure 6a) and the heights (H) of the “hill” shapes were 30.26 μm and 39.81 μm each (Figure 6b). The difference between the experimental data and the simulated data is that cracks were generated during the experiment (Figure 9), which released a part of the stress and made the shape more spread. It demonstrated that a good agreement occurred between the numerical prediction and the experimental measurement. 

This shows that not only the gasification quantity increased but also the melt quantity gradually increased with the laser ablation time. Under the action of both reverse stamping and the Marangoni effect, more fluid flowed upward and remained to solidify. Therefore, back stamping and the Marangoni effect played important roles during the continuous laser damage process. 

To explain the laser ablation temperature field influence on the laser damage, the curve of temperature changing with the distance from the center during laser irradiation is drawn in Figure 5. It shows that the temperature gradually decreased along the direction away from the center due to the center temperature of the laser beam being the highest. When the center peak temperature reached the vaporization temperature of the Al_2_O_3_ ceramic material, a pit would be formed. With the time increasing, more materials evaporated, which led to the increase of the laser damage hole diameter and depth. Figure 3, Figure 4 and Figure 6 also agree with this trend. 

#### 4.1.2. Stress Field and Laser Damage Morphology

**Figure 7:**
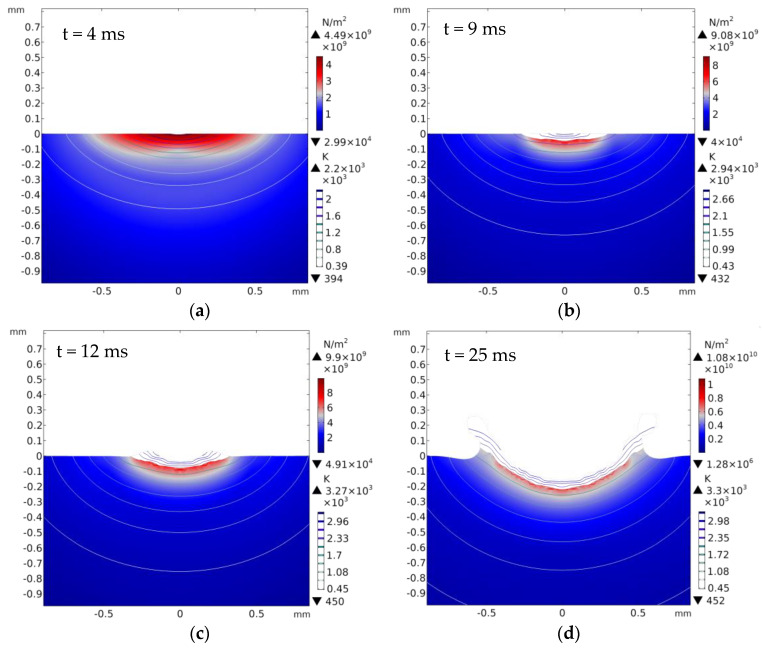
The cloud pictures of stress distributions with different laser ablation times (**a**) t = 4 ms, (**b**) t = 9 ms, (**c**) t = 12 ms, (**d**) t = 25 ms.

**Figure 8:**
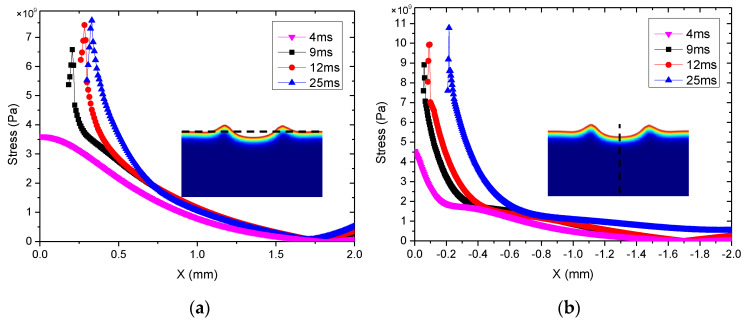
Maximum tensile stress curves with different laser ablation times: (**a**) horizontal (black curve position), (**b**) longitudinal (black curve position).

**Figure 9:**
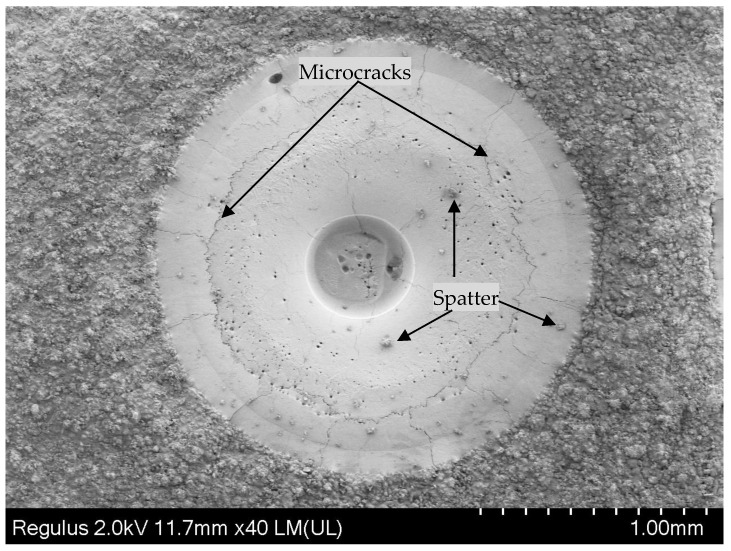
The macrograph of laser damage experiment.

The cloud pictures of stress distribution with different laser ablation times (t = 4, 9, 12, and 25 ms) are demonstrated in Figure 7a–d, where t = 4 ms is the stress distribution before melting. This shows that all of the maximum stress occurred near the hole edge at different times. The mechanism of micro-crack formation after laser ablation was analyzed from two aspects. First of all, the Al_2_O_3_ ceramic materials absorbed laser energy during laser ablation processing, the temperature rose rapidly, and thermal stress was produced around the pits. From reference [33,34], the formula *σ* = *αE*Δ*T*/(1 − *v*) can be obtained, in which *E* is the modulus of elasticity, *α* is the coefficient of linear thermal expansion of the material, and *v* is the Poisson’s ratio. This shows that the laser-induced thermal stress was generated, and the thermal stress was proportional to Δ*T*; that is, the higher the temperature was, the greater the thermal stress was. When the thermal stress reached the strength of the material, micro-cracks occurred. The maximum stress of the simulation was approximately 1700~2500 MPa, which was greater than its tensile strength (350~500 MPa). Consequently, the micro-cracks occurred as shown in Figure 9. Second, according to the first strength theory, the maximum tensile stress was the main factor leading to brittle fracture damage. When the maximum tensile stress reached the tensile strength of the Al_2_O_3_ ceramic coating, the brittle fracture would occur. According to the relationship curve of stress and radial direction in Figure 8, it exceeded the tensile strength (350~500 MPa) and the maximum stress occurred near the hole edge, which was consistent with the location of micro-cracks in Figure 9. 

### 4.2. Mechanism of Laser Ablation Damage

**Figure 10:**
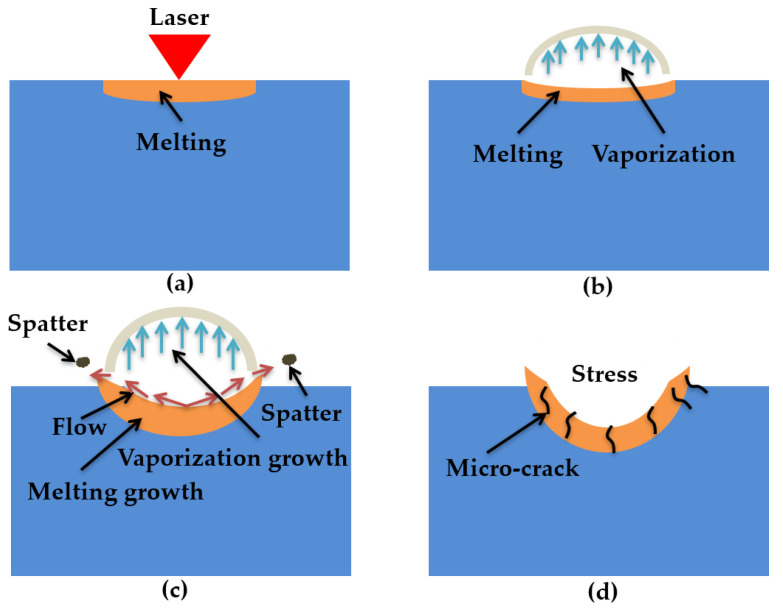
Schematic diagram of laser ablation damage: (**a**) only melting occurred; (**b**) vaporization has just occurred; (**c**) laminar flow and splash; (**d**) micro-cracks produced during laser ablation.

When the laser irradiated the ceramic sample surface, the ceramic absorbed the laser energy (Figure 10a). Because of the Gaussian distribution of the laser, the sample surface quickly reached the material melting point (T_m_) (Figure 5). The material melted and the fluid velocity was low (Figure 3a,b and Figure 10a). Under both the reverse stamping and the Marangoni effect, only minute quantity fluids flowed (Figure 3b). As time went on, the temperature continued to rise to the material vaporization temperature (Figure 5). The material was in two states of melting and gasification (Figure 10b). The fluid velocity and gasification rate increased with time, more fluids flowed up and evaporated, which led to the increase of the laser hole diameter and depth (Figure 3c and Figure 10c), and the ceramic coating was damaged and a pit was formed (Figure 10c). After laser irradiation for 25 ms, the laser was turned off, the molten pool began to cool, the spatters occurred at the same time and finally, the morphology shown in Figure 4, Figure 5 and Figure 9 was formed. On the other hand, the thermal stress produced was caused by the high temperature, leading to micro-cracks (Figure 10d). Moreover, the ceramic material conformed to the brittle fracture strength theory. With the laser ablation time increasing, the tensile stress gradually increased, and cracks were produced when the tensile stress was greater than the material stress (Figure 10d). In conclusion, the laser irradiation damage mechanism included melting, vaporization, spattering, and micro-cracking. Through the above mechanism analysis, we can preliminarily predict the damage of alumina coating by laser irradiation, which provides theoretical basis for laser weapon protection work and theoretical support for future research on crack suppression. 

## 5. Conclusions

1. The damage mechanism of laser ablation on the coating surface included melting, gasification, spattering, and micro-cracking. The main melting damage and spatter factors were surface tension and the Marangoni effect. The main cause of gasification was back stamping, and the main cause of micro-cracks was thermal stress.
2. The temperature-field, fluid-field, and morphology evolution of the damage pit irradiated by the laser were simulated via COMSOL. It determined that the temperature near the laser ablation center (x = 0 mm) was highest and the fluid velocity was fastest near the edge, and it decreased gradually as the distance from the center increased. Additionally, the sizes (De, Di_1, Di_2, H) of the laser damage pit demonstrated that the simulation result agreed with the experiment result.
3. The stress field of the alumina coating irradiated by the laser was simulated via COMSOL. The thermal stress (3500~10,000 MPa) produced by the laser irradiation was much greater than the tensile strength (350~500 MPa) of the material itself, which led to hot cracks and brittle fractures. The morphology after the laser irradiation experiment indicated that micro-cracks occurred on the surface.

## Figures and Tables

**Table 1:** Thermo-physical properties of TC4.

Property	TC4	Value
Density (kg/m^3^)	*ρ _TC4_*	4.51 × 10^3^
Specific heat capacity (J/(kg∗K))	*C_P_*-*_TC4_*	536
Heat conductivity (W/(m∗K))	*k _TC4_*	6.4

**Table 2:** Thermo-physical properties of Al_2_O_3_ ceramic material [25,26,27,28,29,30].

Thermo-Physical Properties of Al_2_O_3_	Value
Density (*ρ_s_*/*ρ_l_*, kg/m^3^)	3800
Density (*ρ_g_*, kg/m^3^)	1.3
Melting temperature (T_m_, K)	2306
Vaporization temperature (T_v_, K)	3250
Latent heat of fusion (L_m_, J/kg)	1.06743 × 10^6^
Latent heat of vaporization (L_v_, J/kg)	1.0665 × 10^6^
Viscosity (*u_l_*/*u_g_*, Pa∗s)	0.069/0.000024
Heat transfer coefficient (h, W/(m^2^∗K))	10
Surface tension gradient (ζ, N/(m∗K))	‒8.2 × 10^−5^
Absorptivity (*A*)	0.25
The emissivity of alumina (ε)	0.7
Stefan–Boltzmann constant (k_b_, W/(m^2^∗K^4)^)	5.67 × 10^−8^
Gas constant (R, J/(mol∗K))	8.31
Poisson’s ratio (*v*)	0.245
Tensile strength *(δ*, MPa)	350–500
Half-width of the temperature curve (ΔT, K)	30

**Table 3:** Laser experiment parameters.

Laser Ablation Parameters	Value
Laser power (*Q*, W)	500
Laser the beam radius (*r_spot*, mm)	0.8
Wavelength (*λ*, nm)	1064
Laser ablation time (t_on_, ms)	25
